# Retracted: The Parenteral Vitamin C Improves Sepsis and Sepsis-Induced Multiple Organ Dysfunction Syndrome via Preventing Cellular Immunosuppression

**DOI:** 10.1155/2020/8462563

**Published:** 2020-09-04

**Authors:** Mediators of Inflammation

*Mediators of Inflammation* has retracted the article titled “The Parenteral Vitamin C Improves Sepsis and Sepsis-Induced Multiple Organ Dysfunction Syndrome via Preventing Cellular Immunosuppression” [1] due to figure duplication between articles by the same authors.

Figure duplication concerns were raised to our attention and then noted on PubPeer [2]. A reassessment of the article concluded that a number of panels in Figure 2(g) of [1] were duplicates of panels in Figure 1 of [3]. Duplications were also identified in another article [4], where the fourth panel of Figure 5(e) in [1] duplicates the third panel of Figure 8(b) in [4].

The authors did not provide a satisfactory response and the article is therefore being retracted with the agreement of the Chief Editor. The authors do not agree to the retraction.
